# Management and Outcomes of Post-Intubation Tracheal Stenosis After COVID-19: A Propensity Score-Matched Study

**DOI:** 10.1093/icvts/ivaf282

**Published:** 2026-02-02

**Authors:** Arman Hasanzade, Fariba Ghorbani, Amir Ali Mahboobipour, Morteza Yaqini, Saviz Pejhan, Mohammad Behgam Shadmehr

**Affiliations:** Tracheal Diseases Research Center, National Research Institute of Tuberculosis and Lung Diseases, Shahid Beheshti University of Medical Sciences, Tehran 1956944413, Iran; Lung Transplantation Research Center, National Research Institute of Tuberculosis and Lung Diseases, Shahid Beheshti University of Medical Sciences, Tehran 1956944413, Iran; Tracheal Diseases Research Center, National Research Institute of Tuberculosis and Lung Diseases, Shahid Beheshti University of Medical Sciences, Tehran 1956944413, Iran; Tracheal Diseases Research Center, National Research Institute of Tuberculosis and Lung Diseases, Shahid Beheshti University of Medical Sciences, Tehran 1956944413, Iran; Tracheal Diseases Research Center, National Research Institute of Tuberculosis and Lung Diseases, Shahid Beheshti University of Medical Sciences, Tehran 1956944413, Iran; Tracheal Diseases Research Center, National Research Institute of Tuberculosis and Lung Diseases, Shahid Beheshti University of Medical Sciences, Tehran 1956944413, Iran; Tracheal Diseases Research Center, National Research Institute of Tuberculosis and Lung Diseases, Shahid Beheshti University of Medical Sciences, Tehran 1956944413, Iran

**Keywords:** post-intubation tracheal stenosis, tracheal stenosis, COVID-19, SARS-CoV-2, tracheal resection and anastomosis, intubation

## Abstract

**Objectives:**

The high intubation rate due to COVID-19 infection has increased the prevalence of post-intubation tracheal stenosis (PITS). We aimed to compare PITS induced by COVID-19 infection with PITS from non-COVID-19 aetiologies.

**Methods:**

This cohort study utilized PITS data collected prospectively between January 2018 and May 2023. A total of 337 PITS patients were identified, and those with direct neck trauma, burn inhalation injuries, and a prior history of tracheal surgery were excluded. Sixty-one COVID-19-related (CR) and 243 non-COVID-19-related (NCR) patients were compared before and after propensity score matching (PSM) including demographics, comorbidities, intubation duration, clinical presentation, stenosis characteristics, the need for resectional airway surgery, and outcomes.

**Results:**

Before matching, CR patients were older, more diabetic, less smokers, and they experienced longer intubation durations. After PSM, 59 CR and 59 NCR PITS patients were included for comparison. Although no statistically significant differences were observed in stenosis, CR PITS patients required significantly more airway resection (81.4%) for definitive management than NCR (42.4%), *P*-value < .001. Postoperative complications, recurrence, and outcomes revealed no significant differences after PSM.

**Conclusions:**

Given the increased need for surgical resection in CR PITS patients, thoracic surgeons should consider the increased likelihood of conservative therapy failure in them. Considering this finding and that no statistically significant difference was observed in postoperative outcomes in our study, we recommend a sooner decision for resectional airway surgery in CR than NCR PITS patients.

## Graphical Abstract

**Figure:**
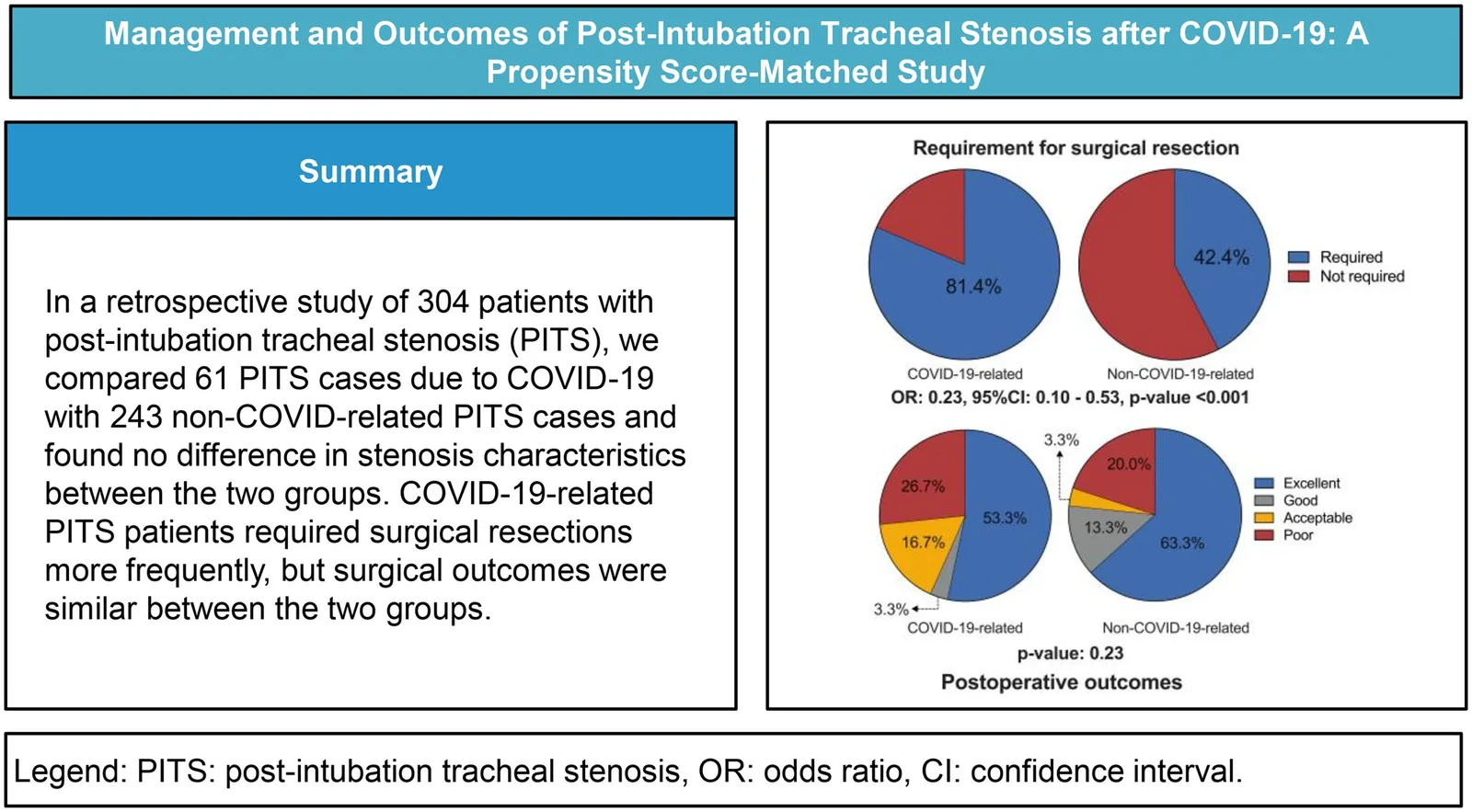

## INTRODUCTION

Post-intubation tracheal stenosis (PITS) is the primary cause of laryngotracheal stenosis, induced by prolonged intubation.^1^ The reported incidence of laryngotracheal stenosis following intubation varies widely (6%-21%).^2^^,^^3^ The estimated annual incidence rate for symptomatic PITS is reported to be approximately 4.9 cases per million.^4^

Acute respiratory distress syndrome was the main complication of severe acute respiratory syndrome coronavirus-2 (SARS-CoV-2), known as COVID-19, often necessitating invasive mechanical ventilation. Early in the pandemic, intubation rates ranged from 9.8% to 33% overall^5^^,^^6^ and were even higher among intensive care unit (ICU)-admitted patients.^7^ However, later studies reported a decline in the need for invasive ventilation.^8^ Nevertheless, the high frequency of intubation in COVID-19 patients was associated with an increased prevalence of PITS.^9^

Although this elevated prevalence of PITS was a consequence of the increased number of intubations, it may also raise concerns that COVID-19 infection itself could increase the risk of stenosis. This may be due to a longer intubation time period required for COVID-19 patients. It may also be caused by more endotracheal or tracheostomy tube cuff inflation to decrease the load of virus contamination.^10–12^ There are also some concerns that the virus itself may cause additional injury, as cases of tracheal stenosis due to COVID-19 infection without intubation have also been reported.^13^^,^^14^ Few studies have evaluated the incidence of PITS following COVID-19-related (CR) intubation, reporting a wide range of 3.3%-40%.^10–12^

Whether the increased risk of PITS is directly related to COVID-19 infection or solely due to high intubation rates, PITS as a result of COVID-19 infection has not been properly evaluated. This study aimed to evaluate stenosis following CR intubation and compare it with non-COVID-19 PITS cases.

## MATERIALS AND METHODS

### Study design

This cohort was conducted at Masih Daneshvari Hospital, a tertiary chest referral centre, to evaluate aspects of PITS in patients intubated due to COVID-19 pneumonia and to compare them with those intubated for non-COVID-19-related (NCR) causes. The primary objective was to compare the need for tracheal resection and anastomosis (TRA) in PITS patients induced by COVID-19 (CR) with NCR cases. Secondary objectives included the comparison of the demographics, comorbidities, intubation period, clinical presentations, stenosis characteristics, and postoperative outcomes. We used the ALBORZ database, which has prospectively recorded data on all patients with laryngotracheal stenosis treated at this centre since January 1994.

Data of all CR PITS patients between September 2020 and May 2023 were extracted. For the NCR group, we enrolled PITS patients admitted between January 2018 and May 2023. In the NCR group, patients intubated due to multiple trauma were excluded since it was unclear whether there was simultaneous blunt trauma to their neck or chest. Patients in both groups were excluded if, in addition to intubation, there were any other potential causes of stenosis, such as direct neck trauma or a prior history of TRA.

### Ethical statement

This study was approved by the Ethics Committee of the National Research Institute of Tuberculosis and Lung Diseases (IR.SBMU.MSP.REC.1401.27) and conducted in line with the Declaration of Helsinki. Data were extracted anonymously from the ALBORZ database, where patient consent is obtained at registration. As no identifiable information was used, additional consent was waived per ethics committee policy.

### Matching and statistical analysis

Before propensity score matching (PSM), CR and NCR patients were compared in their baseline characteristics to identify all possible covariates. Factors found to differ between the 2 groups included age, gender, intubation duration, diabetes, and smoking status. PSM was conducted on a 1:1 basis without replacement, using a caliper of 0.3 times the SD of the propensity score, based on these variables, to ensure optimal matching performance. After matching, we re-assessed the groups for all the mentioned covariates to confirm matching accuracy. The statistical analysis was performed using SPSS v27.0.1, with the R plugin and an auxiliary tool used for PSM.

CR and NCR patients were compared based on PITS presentation and stenosis characteristics. First bronchoscopy reports by thoracic surgeons were reviewed to assess stenosis location, grade, and length. Grading followed the Myer-Cotton classification: I (0%-50%), II (50%-75%), III (75%-99%), and IV (complete obstruction). We also evaluated the rate of patients requiring surgical resection, including those who underwent TRA at our centre or elsewhere, or received tracheostomy/T-tube placement due to contraindications for surgery or anaesthesia.

For the postoperative outcomes, only patients who underwent TRA at our centre were included, and a new PSM was performed with a similar strategy. The evaluated outcomes included postoperative complications (wound infection, dehiscence, haematoma/seroma, vocal cord paralysis, granulation tissue formation, and recurrence). In cases of recurrence unresponsive to several bronchoscopic dilatations, patients underwent reoperation or tracheostomy/T-tube placement, either as a bridge to reoperation or as a permanent treatment for end-stage airways.

Patients’ outcomes were evaluated based on their symptoms. The outcome was considered “Excellent” if the patient had no dyspnoea or voice changes after TRA and required no further postoperative intervention. If the patient required any postoperative intervention (such as bronchoscopic dilatation) but ultimately had no dyspnoea or voice alteration, the outcome was classified as “Good.” If the patient had a husky voice and/or exertional dyspnoea, the outcome was considered “Acceptable.” The outcome was considered “Poor” if patient required tracheostomy/T-tube, or reoperation. All patients were followed by clinic visits and/or bronchoscopy. Those who did not come back to us for follow-up were contacted by telephone.

Before matching, chi-square tests were used for categorical variables and independent *t*-tests for quantitative ones. After matching, CR and NCR pairs were compared using McNemar’s test for binary variables, paired *t*-tests for quantitative variables, and generalized estimating equation for multinomial variables. Results were reported with *P*-values, mean differences, odds ratios (ORs), and 95% confidence intervals (CIs). To assess the strength of our findings, we used G*Power version 3.1.9.6 to calculate the statistical power of the comparison between the 2 groups regarding our main objective, surgical requirement, both before and after matching.

## RESULTS

### Study population

A total of 337 PITS patients were entered. Thirty-three were excluded (17 prior TRA, 15 direct blunt neck trauma or burn inhalation, 1 concomitant congenital stenosis). Of the remaining patients, 61 underwent intubation due to COVID-19 infection.

As shown in **Table 1**, there were significant differences between 2 groups regarding age, smoking, and diabetes, while gender distribution and heart failure were similar. Intubation duration was longer in the CR patients, with an average of 24.4 ± 24.7 days, compared to 15.5 ± 17.3 days (*P*-value = .01). Tracheostomy had been performed more in CR patients (*P*-value = .26).

**Table 1.** Baseline Characteristics

	Before matching			After matching		
	CR (*n* = 61)	NCR (*n* = 243)	*P*-value	CR (*n* = 59)	NCR (*n* = 59)	*P*-value
**Demographic**						
Age (SD)	46.3 (13.8)	35.1 (16.9)	<.001	46.3 (12.8)	47.4 (19.3)	.58
Male (%)	32 (52.5)	146 (60.1)	.31	31 (52.5)	30 (50.8)	1.00
**Comorbidities (%)**						
Diabetes	18 (29.5)	21 (8.6)	<.001	17 (28.8)	12 (20.3)	.18
Heart failure	1 (1.6)	10 (4.1)	.70	1 (1.7)	6 (10.2)	.12
Smoking	4 (6.6)	52 (21.4)	.006	4 (6.8)	4 (6.8)	1.00
**Intubation duration (SD)**	24.4 (24.7)	15.5 (17.3)	.01	24.5 (24.8)	19.6 (24.9)	.10
**Tracheostomy (%)**	22 (36.1)	69 (28.6)	.26	22 (37.3)	22 (37.3)	1.00
**Asymptomatic duration (SD)**	26.0 (53.1)	10.9 (15.5)	.03	26.3 (53.5)	9.1 (14.9)	.02

Abbreviations: CR, COVID-19-related; NCR, non-COVID-19-related.

After determining these disparities, PSM was performed based on the age, gender, intubation duration, diabetes, and smoking status. After PSM, there were no differences between 2 groups regarding the age, gender, comorbidities, intubation duration, or tracheostomy status.

Exertional dyspnoea and stridor were the most common symptoms in both groups before and after PSM. As shown in **Table S1**, there were no differences between the 2 groups regarding the symptoms, except for hoarseness only after PSM. However, the duration from extubation to symptom presentation (asymptomatic duration) was significantly longer in CR patients (*P*-value = .03), which persisted after PSM (*P*-value = .02) (**Table 1**).

### Bronchoscopic characteristics

The stenosis characteristics were not statistically different between the 2 groups before and after PSM (**Table 2**).

**Table 2.** Stenosis Characteristics

	Before matching					After matching				
	CR (*n* = 61)	NCR (*n* = 243)	MD	95% CI	*P*-value	CR (*n* = 59)	NCR (*n* = 59)	MD	95% CI	*P*-value
**Length of stenosis (SD)**	28.5 (10.3)	29.6 (7.7)	1.05	−1.77 to 3.87	.46	28.6 (10.5)	29.3 (8.7)	−0.69	−4.59 to 3.20	.72
**Vocal cord to stenosis distance (SD)**	24.8 (13.3)	24.4 (10.5)	−0.32	−3.96 to 3.32	.86	24.9 (13.4)	25.9 (12.7)	−1.02	−5.68 to 3.62	.66
**Stenosis to carina distance (SD)**	56.8 (16.8)	60.7 (13.4)	3.93	−0.69 to 8.57	.09	56.8 (17.0)	60.2 (15.6)	−3.01	−8.92 to 2.11	.22
**Airway diameter at stenosis (SD)**	6.5 (2.1)	6.3 (2.2)	−0.20	−0.82 to 0.40	.50	6.5 (2.1)	6.4 (2.1)	0.11	−0.73 to 0.96	.79
**Grading** (%)										
Mild	3 (4.9)	15 (6.2)			.89	3 (5.1)	7 (11.9)			.09
Moderate	5 (8.2)	25 (10.3)				5 (8.5)	10 (16.9)			
Severe	48 (78.7)	177 (72.8)				46 (78.0)	37 (62.7)			
Complete obstruction	5 (8.2)	26 (10.7)				5 (8.5)	5 (8.5)			
**Location** (%)										
Tracheal	32 (52.5)	122 (50.2)			.96	31 (52.5)	33 (55.9)			.58
Subglottic	12 (19.7)	50 (20.6)				11 (18.6)	11 (18.6)			
Tracheal and subglottic	17 (27.9)	71 (29.2)				17 (28.8)	15 (28.8)			

Abbreviations: CR, COVID-19-related; MD, mean differences; NCR, non-COVID-19-related.

### Stenosis management

TRA was indicated in 49 (80.3%) CR patients and 139 (57.2%) NCR patients (*P*-value = .001). After PSM, this difference became even more noticeable, 81.4% CR vs 42.4% NCR, *P*-value < .001 (**Table 3**, Management). For this comparison, the estimated statistical power was 0.92 before matching, and increased to over 0.95 after matching.

**Table 3.** Post-intubation Tracheal Stenosis Management and Outcomes

Management										
	Before matching					After matching				
	CR (*n* = 61)	NCR (*n* = 243)	OR	95% CI	*P*-value	CR (*n* = 59)	NCR (*n* = 59)	OR	95% CI	*P*-value
**Surgical resection was required (%)**	49 (80.3)	139 (57.2)	0.39	0.22-0.71	.001	48 (81.4)	25 (42.4)	0.23	0.10-0.53	<.001

Postoperative outcomes										
	Before matching					After matching				
Complication	CR (*n* = 40)	NCR (*n* = 89)	OR	95% CI	*P*-value	CR (*n* = 31)	NCR (*n* = 31)	OR	95% CI	*P*-value
Wound infection (%)	7 (17.5)	5 (5.6)	0.48	0.84-3.39	.04	5 (16.1)	0	—	—	.06
Granulation tissue (%)	12 (30.0)	17 (19.1)	0.67	0.39-1.15	.17	8 (25.8)	5 (16.1)	0.57	0.16-1.95	.54
Dehiscence (%)	5 (12.5)	7 (7.9)	0.71	0.34-1.48	.41	4 (12.9)	2 (6.5)	0.33	0.03-3.20	.62
Seroma/haematoma (%)	2 (5.0)	5 (5.6)	1.09	0.32-3.62	1.00	1 (3.2)	1 (3.2)	1.00	0.06-15.9	1.00
Vocal cord paralysis (%)	1 (2.5)	5 (5.6)	1.90	0.31-11.6	.66	1 (3.2)	1 (3.2)	1.00	0.06-15.9	1.00
Recurrence (%)	9 (22.5)	9 (10.1)	0.55	0.32-0.96	.09	8 (25.8)	5 (16.1)	0.50	0.12-1.99	.50
**Final outcome (%)** ^a^	**CR (*n *= 39)**	**NCR (*n* = 86)**			***P*-value**	**CR (*n* = 30)**	**NCR (*n* = 30)**			***P*-value**
Excellent	23 (59.0)	64 (72.7)			.01	16 (53.3)	19 (63.3)			.23
Good	1 (2.6)	11 (12.5)				1 (3.3)	4 (13.3)			
Acceptable	5 (12.8)	3 (3.4)				5 (16.7)	1 (3.3)			
Poor	10 (25.6)	10 (11.4)				8 (26.7)	6 (20.0)			

Abbreviations: CR, COVID-19-related; NCR, non-COVID-19-related; OR, odds ratio.

a Long-term follow-up was unavailable for 2 NCR patients before propensity score matching (PSM) and 1 after, leaving 29 CR-NCR pairs for post-PSM analysis.

While the history of tracheostomy did not differ between groups before or after matching, it was not included as a covariate in the PSM. Therefore, we were concerned whether it could affect the requirement for surgical resection. To address this, we divided our cohort based on tracheostomy history. As shown in **Table 4**, surgical resection was more frequently required in CR patients, both in those with and without a history of tracheostomy. Also, to ensure that the higher requirement for surgical resection did not change over time since the start of the COVID-19 pandemic, we modelled the number of days from pandemic onset to intubation using binary logistic regression. No significant temporal change was observed (Exp(β) = 1.00, *P *= .905).

**Table 4.** Tracheostomy History and the Requirement for TRA

	*N*	TRA was required	OR	CI	*P*-value
**Without tracheostomy history**	213				
CR	39	33 (84.6)	0.75	0.64-0.89	.013
NCR	174	111 (63.8)			
**With tracheostomy history**	91				
CR	22	16 (72.7)	0.55	0.38-0.81	.008
NCR	69	28 (40.6)			

Abbreviations: CR, COVID-19-related; NCR, non-COVID-19-related; OR, odds ratio; TRA, tracheal resection and anastomosis.

Of the 188 candidates for surgery, 140 (41 CRs, 99 NCRs) underwent TRA at our centre. One CR and 10 NCR patients were lost to follow-up. PSM was performed again for the remaining patients, resulting in 31 matched pairs.

There was no difference in postoperative complications (**Table 3**, Postoperative outcomes), except for a higher occurrence of wound infection in the CR group. This difference was not significant after PSM.

Recurrence (restenosis unresponsive to one or a few attempts of bronchoscopic dilatations) was observed in 9 (22.5%) of CR and 9 (10.1%) of NCR patients before PSM (*P*-value = .09) and in 8 CR and 5 NCR patients after PSM (*P*-value = .50).

Of the 18 patients with recurrence, one had reoperation without prior tracheostomy/T-tube and remained well with no restenosis. Of the other 17 with tracheostomy/T-tube, 7 were successfully decannulated, 4 had re-operations, and 6 remained cannulated at the end of our study follow-up. Among the 4 re-operated, 2 recovered without further intervention, while 2 required re-tracheostomy/T-tube but were eventually decannulated. Details are shown in **Figure 1**.

**Figure 1.**
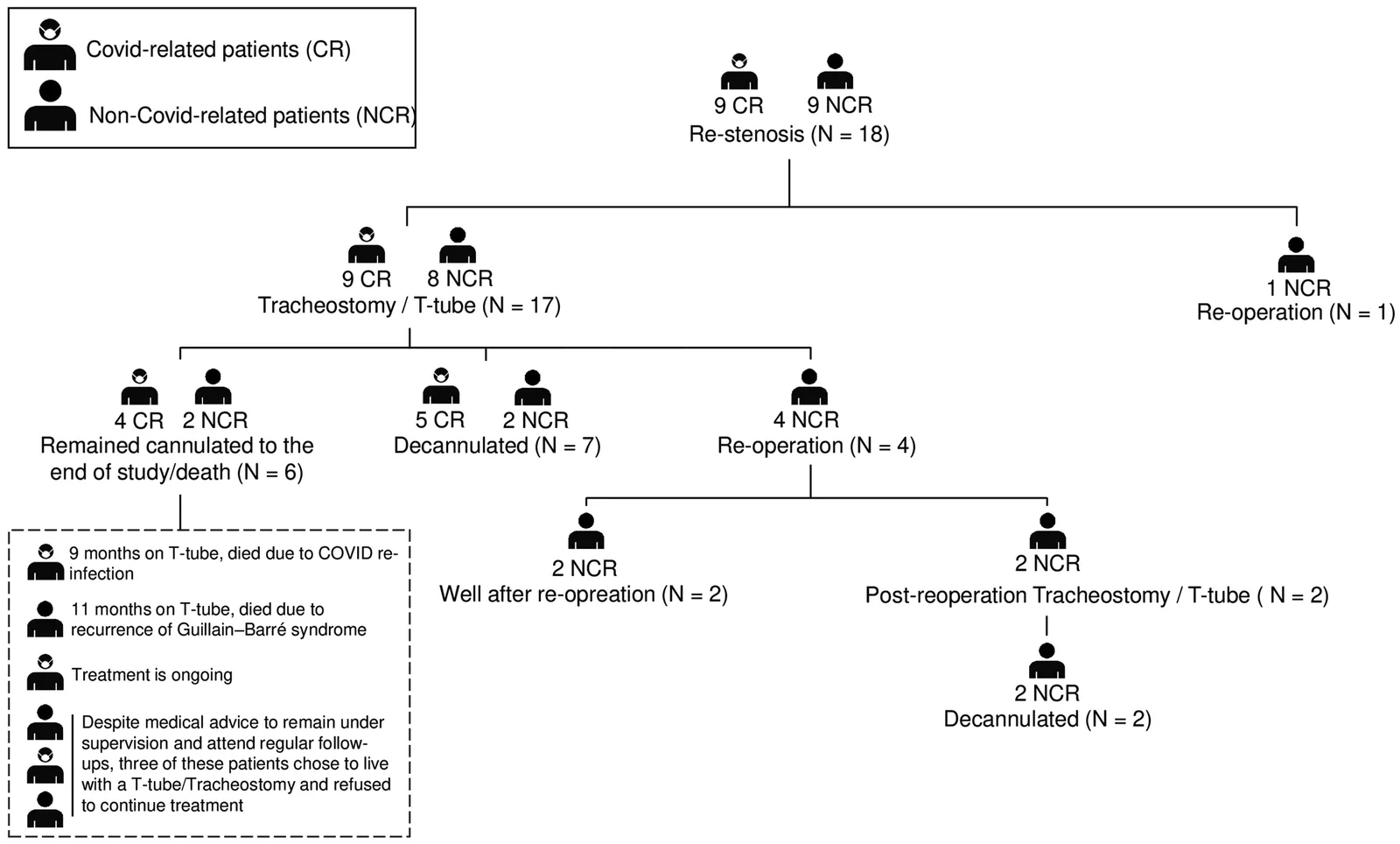
Management of Restenosis Unresponsive to Bronchoscopic Dilatation

The decannulation rate was assessed among 17 patients who underwent tracheostomy/T-tube insertion. Decannulation was successful in 55.6% CR and 22.2% NCR patients (OR: 1.78, CI: 0.73-4.35, *P*-value = .33).

From the 40 CR and 89 NCR operated patients, 2 NCR patients (1 from matched patients) were lost to follow-up. Among the remaining patients, significant differences in outcomes were observed before PSM, as it shown in **Table 3**. However, comparing outcomes after PSM revealed no significant differences (*P*-value = .23) (**Table 3**).

In addition to the 2 mortalities described in **Figure 1**, one patient died on postoperative day 8 due to myocardial infarction, and another patient died 1 year after surgery following an asthma attack and respiratory arrest.

## DISCUSSION

We evaluated PITS in patients intubated for COVID-19 and compared them to those intubated for non-COVID-19 reasons. CR patients had significantly longer intubation durations, likely due to delayed tracheostomy to reduce viral transmission risk. They were also older and more likely to have diabetes, while smoking was more common in NCR patients.

The use of larger endotracheal tubes and higher cuff pressures, which were assumed to minimize viral transmission, may have influenced the increased incidence of PITS in COVID-19 cases.^15^^,^^16^ Prolonged intubation periods and delayed tracheostomy are additional related factors. While tracheostomy is typically recommended 7-14 days after intubation to decrease the long-term complications, the higher risk of viral transmission during tracheostomy has led to a median intubation time of 17 days for COVID-19 patients in several reported studies.^5^^,^^6^^,^^11^^,^^15–17^ COVID-19 patients also exhibit a higher rate of reintubation.^5^^,^^15^^,^^16^ Notably, obesity and diabetes, which are established risk factors for both laryngotracheal stenosis and severe COVID-19, may also contribute to the elevated PITS incidence.^15^ Although we did not have access to the detailed intubation data for the patients in this study, we observed a longer duration of intubation in our COVID-19 patients.

PITS symptoms typically emerge once stenosis obstructs 50% of the airway diameter.^18^ This may explain the high prevalence of grade III Cotton-Myer stenosis observed in this study. While we found no significant difference in stenosis severity, more severe stenosis was reported in CR PITS patients.^19^ The duration between extubation and the onset of PITS symptoms was shorter in NCR patients. We assume CR patients attributed their symptoms to prolonged COVID-19 and delayed seeking care, while NCR patients sought medical attention sooner.

The characteristics of stenosis were similar between the 2 groups. These findings contradicted our assumption that COVID-19 patients have more severe stenosis, given the longer intubation times and use of larger endotracheal tube sizes.

Although bronchoscopic dilatational therapy serves as a bridge to TRA for most patients, it may also cure some patients, particularly those with less severe stenosis that has not yet progressed to end-stage fibrotic cartilage destruction.^5^^,^^17^^,^^20^ Here, we performed dilatation by rigid bronchoscopy on both groups. However, 80.3% of CR patients ultimately required TRA, a significantly higher percentage compared to NCR patients (57.2%). This difference persisted even after PSM, indicating that the higher need for TRA in CR patients was not related to covariate factors. This higher requirement for surgical resection is despite the fact that the stenosis was similar between the 2 groups.

The failure of conservative therapy in CR PITS patients has been observed in previous studies as well.^15^^,^^17^ While some studies have highlighted the role of TRA in managing PITS in CR patients,^12^^,^^21^^,^^22^ none have directly compared the necessity of TRA between CR and NCR patients. This issue raises the concern that the COVID-19 virus, independent of endotracheal intubation, may contribute to PITS development or worsen it by long-term effects on the tracheal mucosa. This concern becomes more presumptive knowing that tracheal stenosis in COVID-19 patients with no history of intubation or tracheostomy has also been reported.^13^^,^^14^ Additionally, there are some reports of the COVID-19-induced tracheitis^23^ or the presence of the SARS-CoV-2 virus in the tracheal epithelial cells.^24^^,^^25^

To the best of our knowledge, no prior study has reported a higher necessity for TRA in CR PITS patients. However, some have examined the impact of SARS-CoV-2 on the tracheal mucosa histology, yielding controversial results. A previous study comparing tracheal tissue excised during tracheostomy between COVID-19 and non-COVID-19 patients found no significant differences,^26^ while Fiacchini et al.^27^ observed increased infiltration of innate immune cells in COVID-19 samples. They also identified significant downregulation in 332 of 664 evaluated genes.^27^ Also, Martins et al. observed gene alterations in samples obtained from CR PITS patients. They found dysregulation in retinol metabolism genes that may result in hyper-granulation and hyperproliferation of tracheal mucosa, leading to tracheal stenosis.^28^ While they did not compare with NCR cases, Lyberis et al. compared CR and NCR PITS patients, reporting higher IgG deposits and fibrosis in CR PITS patients and noted that IgG infiltration was observed only in CR cases.^9^

None of the above-mentioned studies provides an answer to why PITS in CR patients require more TRA. Interestingly, studies on lung samples showed that SARS-CoV-2 can cause microvascular injury and necrosis.^18^^,^^24^^,^^29^ This effect of the virus might relate to the higher requirement for surgical resection. Despite the unclear underlying reasons, we propose 2 hypotheses that may explain the increased need for surgical resection. First, we know that those patients with mild to moderate PITS who were treated with several attempts of bronchoscopic interventions did not ultimately achieve a normal tracheal diameter at the end of treatment. However, with persistent mild tracheal stenosis, they experienced no dyspnoea during their normal daily activities. Therefore, they were not recommended for surgery. We believe that since some of the CR PITS patients had some degree of residual lung parenchymal diseases, they could tolerate a lesser degree of tracheal stenosis to remain asymptomatic during routine daily activities, and therefore required more resectional airway surgery.

In addition, by operating on numerous patients with CR organizing empyema, we observed more advanced fibrosis than in NCR empyema. Therefore, as our second hypothesis, we suppose that CR PITS may be more fibrotic, rather than inflammatory in nature.

In our patients, postoperative evaluation indicated no statistically significant differences in the complications rates between the 2 groups, except for wound infections, which were more common in the CR group, only before PSM. Very few studies have evaluated the rates of postoperative complications between CR and NCR patients. A previous study demonstrated that TRA in CR patients is associated with a higher rate of reintubation, postoperative ICU hospitalization, and more postoperative dysphonia. However, no difference regarding the restenosis was found.^18^ Our evaluation indicated better treatment outcomes in the NCR group; although, this superiority did not persist after the PSM analysis.

Our study has some limitations. Most of these patients were referred to our centre for the treatment of PITS, while they were intubated elsewhere. Therefore, details about the intubation history were not fully available. However, even if there was a difference in the intubation history, the 2 groups had similar stenosis characteristics, and yet CR patients required surgical resection more frequently. We also compared COVID-19 cases with patients intubated due to various other aetiologies. While we attempted to exclude intubation causes that were noticeably different from those in COVID-19 cases, there were still some differences. Another limitation is that bronchoscopies and surgeries were performed by different surgeons with varying technical expertise. Additionally, as this was in a single-centre study, further evaluation in multicentre studies can provide a more accurate assessment. It is also notable that, although we attempted to minimize the effects of covariates through PSM, there may still be unassessed differences between the 2 groups. Lastly, although the data were prospectively recorded, they were retrieved retrospectively for this study, which may introduce some limitations of retrospective studies.

Regarding the strengths of our study, although several studies have examined PITS caused by COVID-19 intubation, only a limited number have compared PITS resulting from COVID-19 intubation to other aetiologies. To the best of our knowledge, our study represents the largest cohort of patients evaluated in this context. Although a previous study used the PSM method to compare patients, they only evaluated post-TRA outcomes. This is the first study to compare PITS in CR and NCR patients from presentation to the end of treatment using the PSM method.

## CONCLUSION

Our findings suggest that PITS caused by COVID-19 intubation have similar characteristics to PITS related to NCR intubations. We found that PITS in CR patients required TRA more commonly. Therefore, thoracic surgeons should consider the higher possibility of conservative therapy failure in these patients. Considering that we found no statistically significant differences in postoperative outcomes, we recommend a sooner decision for resectional airway surgery in CR than NCR PITS patients.

## Supplementary Material

ivaf282_Supplementary_Data

## Data Availability

The data are available from the corresponding author upon request.
