# Supplementary material for: Management and Outcomes of Post-Intubation Tracheal Stenosis After COVID-19: A Propensity Score-Matched Study
Source: Interdiscip Cardiovasc Thorac Surg. 2026 Feb 2;41(2):ivaf282. doi: 10.1093/icvts/ivaf282 (PMC13742455; doi:10.1093/icvts/ivaf282)
Supplement: Supplementary file 1 [file ivaf282_supplementary_data.docx]

**Supplementary Data**

**Supplementary Table 1. PITS Presentation**

|  | **Before Matching** | | | **After Matching** | | |
| --- | --- | --- | --- | --- | --- | --- |
|  | CR  (n = 61) | NCR  (n = 243) | P-Value | CR  (n = 59) | NCR  (n = 59) | P-Value |
| **Symptoms** |  |  |  |  |  |  |
| Dyspnea at rest (%) | 38 (62.3) | 148 (60.9) | 0.88 | 37 (62.7) | 34 (57.6) | 0.72 |
| Exertional dyspnea (%) | 48 (78.7) | 211 (86.8) | 0.11 | 47 (79.7) | 52 (88.1) | 0.35 |
| Stridor (%) | 46 (75.4) | 185 (76.1) | 0.86 | 45 (76.3) | 35 (59.3) | 0.06 |
| Cough (%) | 29 (47.5) | 157 (64.4) | 0.01 | 28 (47.5) | 35 (59.3) | 0.24 |
| Sputum (%) | 25 (41.0) | 91 (37.4) | 0.65 | 24 (40.7) | 16 (27.1) | 0.18 |
| Hoarseness (%) | 31 (50.8) | 101 (41.5) | 0.19 | 31 (52.5) | 21 (35.6) | 0.052 |
| Hemoptysis (%) | 0 | 12 (4.9) | 0.13 | 0 | 2 (3.4) | 0.50 |
